# New Routes for Refinery of Biogenic Platform Chemicals Catalyzed by Cerium Oxide-supported Ruthenium Nanoparticles in Water

**DOI:** 10.1038/s41598-017-14373-1

**Published:** 2017-10-25

**Authors:** Tomoo Mizugaki, Keito Togo, Zen Maeno, Takato Mitsudome, Koichiro Jitsukawa, Kiyotomi Kaneda

**Affiliations:** 1 Department of Materials Engineering Science, Graduate School of Engineering Science, Osaka University, 1-3 Machikaneyama, Toyonaka, Osaka, 560-8531 Japan; 2 Research Center for Solar Energy Chemistry, Osaka University, 1-3 Machikaneyama, Toyonaka, Osaka, 560-8531 Japan

## Abstract

Highly selective hydrogenative carbon–carbon bond scission of biomass-derived platform oxygenates was achieved with a cerium oxide-supported ruthenium nanoparticle catalyst in water. The present catalyst enabled the selective cleavage of carbon–carbon σ bonds adjacent to carboxyl, ester, and hydroxymethyl groups, opening new eight synthetic routes to valuable chemicals from biomass derivatives. The high selectivity for such carbon-carbon bond scission over carbon-oxygen bonds was attributed to the multiple catalytic roles of the Ru nanoparticles assisted by the *in situ* formed Ce(OH)_3_.

## Introduction

**Figure 1:**
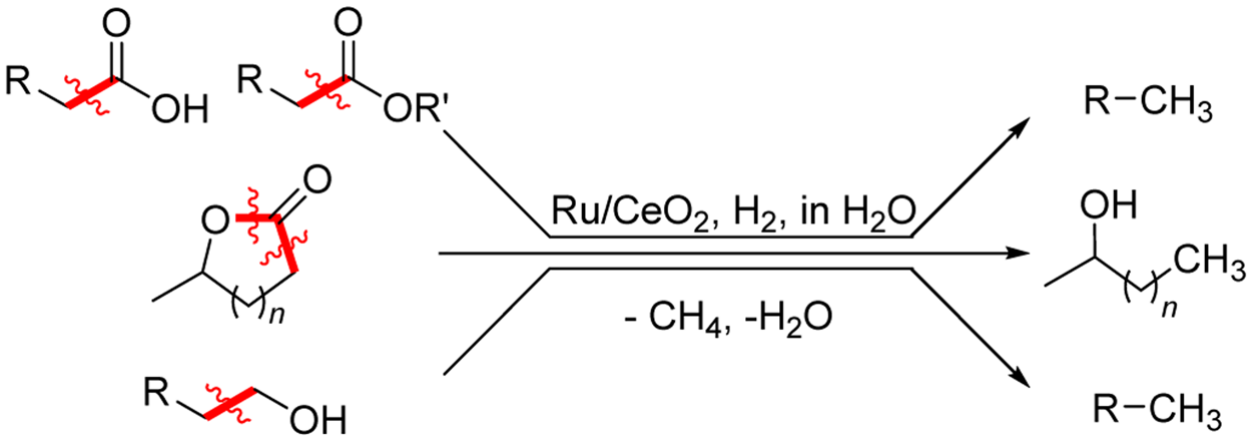
Reductive one-carbon scission of oxygenate compounds such as carboxylic acids, esters, lactones, and primary alcohols catalyzed by Ru/CeO_2_ in water.

Current requirements to reduce carbon dioxide emissions have been the driving force for biorefinery utilizing renewable resources, such as plant biomass, as carbon-neutral feedstocks for commodity chemicals^1–7^. The development of highly efficient catalytic methods would greatly accelerate the utilization of biomass feedstocks in place of fossil resources. To date, much effort has been devoted to the direct carbon–oxygen (C–O) bond cleavage of high-oxygen containing biogenic polyols to produce valuable chemicals by hydrogenolysis and deoxydehydration^8,9^. For example, there are many attempts for the selective hydrogenolysis of glycerol, facilely obtained from fats and oils, to 1,2-propanediol and 1,3-propanediol as the valuable polyester monomers and solvents using the copper- and platinum-based heterogeneous catalysts, respectively^10,11^. On the other hand, selective cleavage of carbon–carbon (C–C) bonds has not yet been widely researched despite its great potential for extending the utility of biomass-derived oxygenates to obtain the desired carbon chain length^12–14^. The existing C–C bond cleavage methods include cracking, hydrocracking, decarbonylation, and decarboxylation^15^. However, these reactions often suffer from low selectivity toward the desired chemicals, limited substrate scope, and high reaction temperatures. Recently, decarboxylation reactions of fatty acids using Ni and Pd catalysts have been reported for the production of biofuels; however, these methods still require harsh reaction conditions^16–18^. Therefore, the development of selective and versatile C–C bond scission catalysts able to work under milder conditions is highly desired to open new routes for industrially important chemicals from a wide range of biomass derivatives^4^. In this work, we found that cerium oxide-supported ruthenium nanoparticles (Ru/CeO_2_) efficiently promote the selective C–C bond scission of levulinic acid (LA) to 2-butanol (2-BuOH) in water. There are many reports on the catalytic transformation of LA to value-added C5 chemicals: e.g. γ-valerolactone (GVL)^19^, 1,4-pentanediol (1,4-PeD)^19^, and 2-methyltetrahydrofuran (MTHF)^20^. However, there are fewer examples of the refinery of LA into valuable C4 chemicals such as 2-butanol through C–C bond scission reactions^21^. The high generality of this method is demonstrated by its broad substrate scope for oxygenated compounds, where the cleavage of C–C bonds occurs chemospecifically at positions adjacent to carboxyl, ester, and hydroxymethyl groups of oxygenated compounds (Fig. 1). Our results provide a simple and green method for the refinery of platform chemicals from biomass, which have been currently produced from petroleum feedstock.

## Results and Discussion

**Figure 2:**
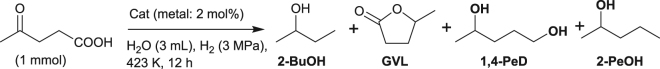
Reductive one-carbon scission of LA in water.

At the outset of our catalytic studies, we chose LA as the model substrate, which is an important platform biomass-derivative leading to a variety of useful chemicals^22,23^. Figure 2 and Table 1 show the results of the hydrogenative decarboxylation of LA using various supported metal catalysts at 423 K and 3 MPa of H_2_. It was found that Ru/CeO_2_ efficiently afforded 2-BuOH in water, currently produced from 1-butene and 2-butene, as the major product in 85% yield (entry 1). The LA consumption and the products formation profiles shown in Figure S1 revealed that conversion of LA rapidly completed within 1 hour, and GVL and 1,4-PeD were formed for 1 hour and three hours, respectively. The yield of 2-BuOH drastically increased after 3 hours duration and the yield reached to 85% after 12 hours with the complete consumption of GVL and 1,4-PeD. The gas chromatography analysis of the gaseous phase after the 12 hours reaction clearly evidenced the formation of CH_4_, while CO and CO_2_ were not detected (Figure S2). These results clearly support that both GVL and 1,4-PeD are not side products but the intermediate products leading to 2-BuOH (vide infra).

**Table 1:** Carbon-carbon scission of LA using various catalysts.

Entry	Catalyst	Solvent	Conv. [%]^[a]^	Yield [%]^[a]^			
				2-BuOH	GVL	1,4-PeD	2-PeOH
1	Ru/CeO_2_	H_2_O	>99	85	0	0	5
2^[b]^	Ru/CeO_2_	H_2_O	>99	81	0	0	6
3	Ru/CeO_2_	2-PrOH	>99	5	69	23	0
4	Ru/CeO_2_	THF	>99	4	70	23	0
5	Ru/CeO_2_	DME	>99	0	77	9	0
6	Ru/HAP	H_2_O	>99	59	16	3	11
7^[c]^	Ru/C	H_2_O	>99	58	0	0	27
8	Ru/ZrO_2_	H_2_O	>99	57	16	3	8
9	Ru/TiO_2_	H_2_O	>99	45	29	5	10
10	Ru/La(OH)_3_	H_2_O	>99	34	27	16	2
11	Ru/HT	H_2_O	>99	17	38	4	1
12	Ru/SiO_2_	H_2_O	>99	6	81	11	1
13	Ru/MgO	H_2_O	>99	6	50	4	0
14	Ru/Al_2_O_3_	H_2_O	>99	1	92	5	0
15	Rh/CeO_2_	H_2_O	>99	<1	66	2	0
16	Pd/CeO_2_	H_2_O	>99	0	77	1	0
17	Ir/CeO_2_	H_2_O	>99	0	71	2	0
18	Pt/CeO_2_	H_2_O	>99	0	68	4	0
19^[d]^	CeO_2_	H_2_O	7	0	0	0	0

^[a]^Analyzed by GC-MS using an internal standard. ^[b]^A 50 mmol-scale reaction (see Supplementary Information for details). ^[c]^Ru/C (Ru 5 wt%, Wako Pure Chemicals) was used. 2-Methyltetrahydrofurane was formed in 8% yield. ^[d]^CeO_2_ 0.1 g was used.

The promising catalysis of Ru/CeO_2_ was compared with those of the Ru-based heterogeneous catalysts. Ru/HAP (hydroxyapatite), Ru/C (activated carbon), Ru/ZrO_2_, Ru/TiO_2_, and Ru/La(OH)_3_ gave moderate yields of 2-BuOH (entries 6–10) and GVL, 1,4-PeD, and 2-PeOH were obtained as the by-products. Ru/HT (hydrotalcite), Ru/SiO_2_, Ru/MgO, and Ru/Al_2_O_3_ returned low yields of 2-BuOH and GVL was the major product (entries 11–14) under the similar conditions shown in entry 1. Among the CeO_2_-supported noble metal catalysts, Ru/CeO_2_ afforded the highest yield of 2-BuOH, while Rh, Pd, Ir, and Pt on CeO_2_ gave much lower yields while GVL was obtained as the major product in 66–77 yields (entry 1 vs. 15–18). The CeO_2_ support did not exhibit any catalytic activity by itself (entry 19). Interestingly, water was found to the best solvent for the production of 2-BuOH; the use of organic solvents such as 2-PrOH (2-propanol), THF (tetrahydrofuran), and DME (dimethoxyethane) resulted in low yields of 2-BuOH (entry 1 vs. 3–5). These results clearly suggest that the combination of Ru, CeO_2_, and water is indispensable to achieve the selective transformation of LA into 2-BuOH. To the best of our knowledge, this is the first report on the highly selective production of 2-BuOH from LA (Table S1). Furthermore, Ru/CeO_2_ worked in a preparative scale reaction; 50 mmol of LA (5.8 g) affording 81% yield of 2-BuOH (entry 2). After the reaction, the Ru/CeO_2_ catalyst was easily separated from the reaction mixture by centrifugation. The Ru/CeO_2_ catalyst could be used for 5 consecutive runs without appreciable loss of the activity and selectivity (Table S6). The ICP-AES analysis clearly showed that any metal leaching did not occur during the reaction, confirming the robustness of the Ru/CeO_2_ catalyst.

Encouraged by the above promising catalysis of Ru/CeO_2_, we applied this catalyst for the selective C–C bond scission of various biogenic oxygenates (Table 2, entries 1–5, 7–10, 12, 15–17, 25, 26). Notably, this catalyst system provided new eight routes for common industrial chemicals from biogenic carboxylic acids, esters, and polyols (entries 2, 5, 7–9, 15–17). Lignocellulose-derived 3-hydroxybutyric acid and its methyl ester selectively afforded 2-PrOH in 81% and 82% yield, respectively (entries 2 and 9), while lignin-derived 4-hydroxybenzoic acid provided 75% yield of cyclohexanol^24^, which is currently manufactured from fossil resources (entry 5)^25^. Methyl and *n*-butyl levulinate selectively afforded 2-BuOH in 82% yield, respectively (entries 7 and 8)^26^. Biogenic polyols were also transformed by the present catalyst system. Reaction of 1,4-PeD, obtained from LA, afforded 2-BuOH in high yield (entry 17). In the case of α, β,ω-triols of 1,2,4-butanetriol and 1,2,6-hexanetriol, the C–C bond scission selectively occurred at the primary hydroxy group distant from the 1,2-dihydroxy moiety and the corresponding 1,2-propanediol and 1,2-pentanediol were obtained in 72% yield (entries 15 and 16). Thus, new routes for common industrial chemicals from biomass-derived oxygenates were successfully established with this Ru/CeO_2_ catalyst. Furthermore, Ru/CeO_2_ afforded higher yields of valuable chemicals for existing transformations of GVL (entry 12), stearic acid (entry 3), lauric acid (entry 4), and their derivatives (entries 10, 25, and 26), compared to the reported catalytic systems which result in lower yields and require organic solvents and/or harsher reaction conditions (Tables S2 and S3).

**Table 2:**
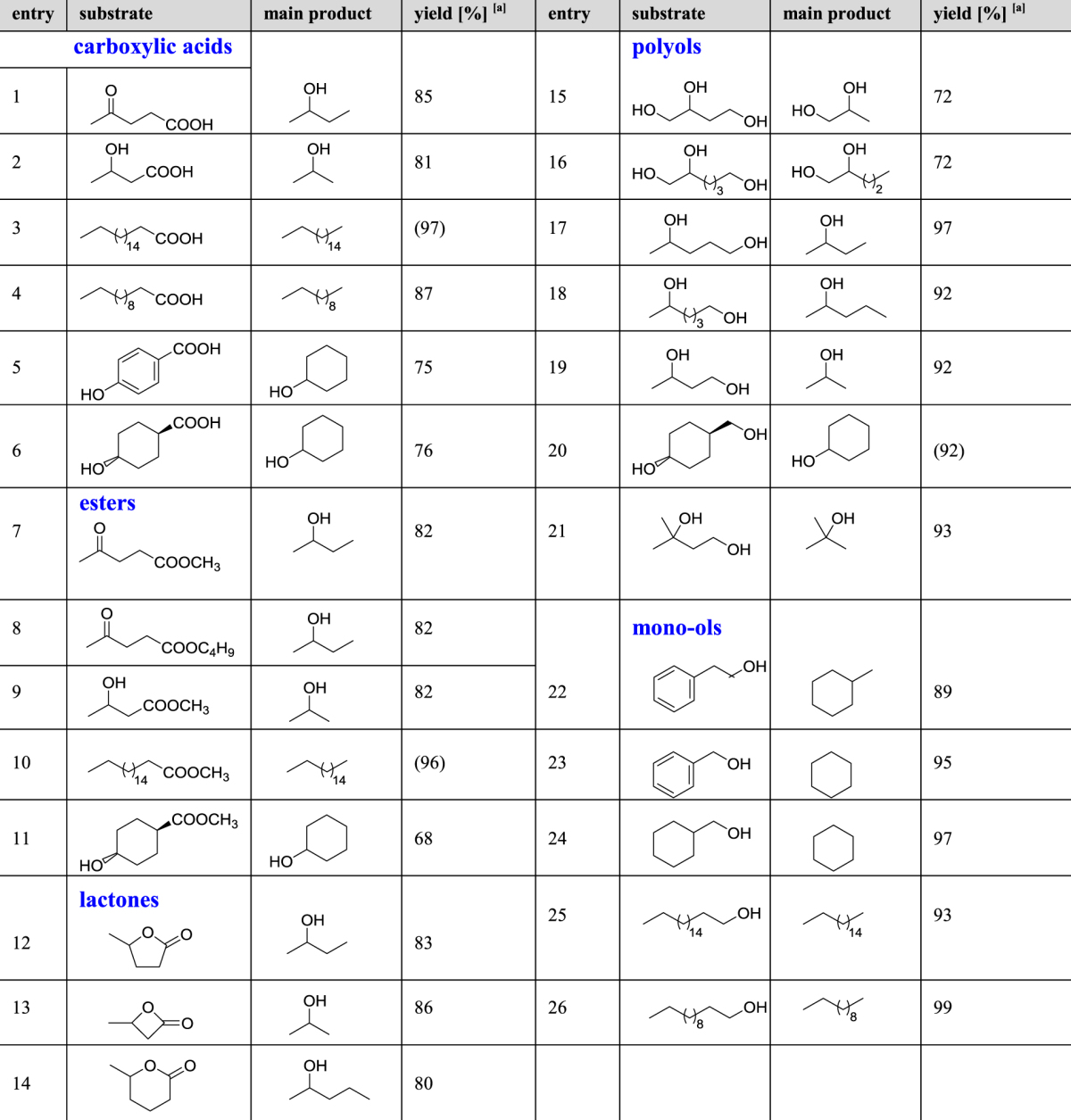
Selective C-C bond scission of various oxygenates catalyzed by Ru/CeO_2_. ^a^Determined by GC-MS using an internal standard technique. The values in parentheses are the isolated yields. See Table S4 and Supplementary Information for details.

**Figure 3:**
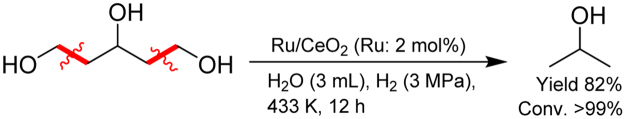
Double dehydroxymethylation of 1,3,5-pentanetriol to 2-propanol.

The high generality of this selective C–C bond scission reaction was also demonstrated using various oxygenated compounds, as listed in Table 2 (entries 6, 11, 13, 14, 18–24). For example, four- and six-membered lactones selectively afforded secondary alcohols in high yield (entries 13 and 14). Cyclohexane was obtained in excellent yield from benzyl alcohol and cyclohexylmethanol (entries 22 and 23)^27^. In the case of diols, the chemospecific cleavage occurred at the C–C bond adjacent to the primary hydroxyl group, while the secondary and tertiary hydroxyl moieties remained intact (entries 18–21). Such a characteristic feature of the Ru/CeO_2_ catalyst regarding the selectivity toward primary hydroxyl groups was confirmed in the double dehydroxymethylation of 1,3,5-pentanetriol to 2-PrOH (Fig. 3). Thus, the Ru/CeO_2_ catalyst is successfully applicable to the C–C bond cleavage of a wide variety of oxygenates not only with short carbon chains but also with long ones and/or aromatic groups.

In order to clarify the origin of the unique catalysis of Ru/CeO_2_ in water, physicochemical analyses of the used Ru/CeO_2_ catalyst were carried out. *In situ* X-ray powder diffraction (XRD) pattern of the used Ru/CeO_2_ catalyst revealed that the CeO_2_ support was reductively hydrothermalized during the reaction to form Ce(OH)_3_ (Figure S3,b vs.c)^28,29^. Furthermore, no diffraction peaks derived from crystalline Ru oxide and Ru metal were observed in the fresh and the used catalysts, suggesting the high dispersity of Ru species, respectively. The Ru K-edge X-ray absorption analysis revealed that the Ru oxide in the fresh catalyst was reduced under the operating conditions to afford metallic Ru nanoparticles (RuNPs) with a mean diameter of ca 3 nm, which agreed well with the sizes obtained from TEM and CO adsorption (Figure S6 and Table S5). The Ce L_3_-edge XANES analysis also confirmed the complete reduction of Ce^4+^ to Ce^3+^ species under the reaction conditions (Figure S5). On the other hand, neither the Ru/CeO_2_ catalysts used in organic solvents nor the CeO_2_ sample without Ru used in water revealed that CeO_2_ was reduced to Ce(OH)_3_ during the reaction. In accordance to the change of the chemical state of Ru/CeO_2_, the FE-SEM analysis also showed the morphology change of the particular CeO_2_ to the nanorods (Figure S6a,b). The Ru dispersion was strongly affected by the support materials and highly dispersed RuNPs were formed only on CeO_2_ (Table S7). Conclusively, highly dispersed RuNPs supported on Ce(OH)_3_ were formed during the reaction in water after the reductive hydrothermal treatment of Ru/CeO_2_.

Taking into account of the above results of the catalytic reactions and the characterization, a reaction pathway for the C–C bond scission of LA to 2-BuOH through multiple catalysis by RuNPs and Ce(OH)_3_ is thus proposed (Figure S7)^16,17,30^. The keto function of LA is readily hydrogenated by RuNPs to give 4-hydroxypentanoic acid (HPA)^31,32^. Then, HPA is further reduced to 4-hydroxypentanal, followed by decarbonylation affords 2-BuOH and Ru-CO species^32^. The resulting CO on RuNPs is hydrogenated to CH_4_ (Figure S2)^33^, completing the catalytic cycle. GVL and 1,4-PeD are reversibly formed as intermediates from HPA and 4-hydroxypentanal, respectively^21^. Hydrolysis of lactone of GVL to HPA is mediated by Ce(OH)_3_^34,35^, and 1,4-PeD is dehydrogenated by RuNPs with assistance of Ce(OH)_3_ at the primary hydroxy group to form 4-hydroxyaldehyde, thereby entering the catalytic cycle^36–38^. Also, the C–C bond scissions of esters including lactones and primary alcohols in Table 2 proceed similar way with the cases of GVL and 1,4-PeD. Overall, RuNPs mediate five successive reactions: reduction of both keto and carboxyl functions, dehydrogenation of a primary hydroxy group, decarbonylation of an aldehyde, and hydrogenation of CO. Ce(OH)_3_ acts as a base to promote (a) the reduction of a carboxyl function, (b) dehydrogenation of a primary hydroxyl group, and (c) hydrolysis of GVL to HPA. Such cooperative catalysis between RuNPs and Ce(OH)_3_ leads to the excellent generality of this selective one-carbon scission of the oxygenated compounds as shown in Table 2. The drastic support effects of Ru catalysts on the 2-BuOH yields were attributed not only to the basicity of the supports, but also to the dispersity of Ru species (Tables 1 and S7).

In summary, selective hydrogenative C–C bond scission over C–O bond of biogenic oxygenates to valuable chemicals was achieved with a Ru/CeO_2_ catalyst in water. The high versatility of the present catalytic system allows the transformation of a variety of carboxylic acids, esters, lactones, and polyols bearing primary hydroxy groups, establishing eight new biorefinery routes. Both the RuNPs and *in situ* generated Ce(OH)_3_ in water are crucial for the highly selective C–C bond cleavage of the presented biomass-derived oxygenates and a wide range of other oxygenated compounds.

## Methods

### Catalyst preparation

The Ru/CeO_2_ catalyst was prepared by the deposition–precipitation method. CeO_2_ (1 g) was added to 50 mL of an aqueous solution of RuCl_3_ (4 mM). After stirring for 1 h, 3 mL of an aqueous NH_3_ solution (28%) was added to the reaction mixture, which was further stirred at 298 K for 12 h. The obtained slurry was filtered and washed with deionized water and dried at 383 K for 12 h, and finally calcined at 573 K for 3 h under static air atmosphere to obtain Ru/CeO_2_ as a blown powder (Ru: 2 wt%). Other metal oxide-supported Ru catalysts (Ru/ZrO_2_, Ru/TiO_2_, Ru/MgO, and Ru/Al_2_O_3_) were also prepared by the deposition–precipitation method.

### Typical reaction procedure

The reactions with levulinic acid were carried out in a 50 mL stainless steel autoclave equipped with a Teflon® vessel. The reactor was charged with 1 mmol of levulinic acid, 0.1 g of Ru/CeO_2_ catalyst, and 3 mL of water; a Teflon®-coated magnetic stir bar was also added. The reactor was sealed, purged three times with H_2_, then pressurized to 3 MPa, heated to 423 K, and stirred at 700 rpm for 12 h. After the reaction, the autoclave was cooled in an ice-water bath and the hydrogen gas was carefully released. The solid catalyst was separated by centrifugation and the reaction mixture was quantitatively analyzed by gas chromatography-mass spectrometry using internal standard method.

## Electronic supplementary material

Supplementary Information
