# Supplementary material for: New Routes for Refinery of Biogenic Platform Chemicals Catalyzed by Cerium Oxide-supported Ruthenium Nanoparticles in Water
Source: Sci Rep. 2017 Oct 25;7:14007. doi: 10.1038/s41598-017-14373-1 (PMC5656575; doi:10.1038/s41598-017-14373-1)
Supplement: Supplementary file 1 — Supplementary Information [file 41598_2017_14373_MOESM1_ESM.pdf]

Supplementary Information

New Routes for Refinery of Biogenic Platform Chemicals Catalyzed by Cerium Oxide-supported Ruthenium Nanoparticles in Water

Tomoo Mizugaki,[1] Keito Togo,[1] Zen Maeno,[1] Takato Mitsudome,[1] Koichiro Jitsukawa[1] and Kiyotomi Kaneda[*1,2]

[1] Department of Materials Engineering Science, Graduate School of Engineering Science, Osaka University,

1-3 Machikaneyama, Toyonaka, Osaka 560-8531 (Japan)

[2] Research Center for Solar Energy Chemistry Osaka University

1-3 Machikaneyama, Toyonaka, Osaka 560-8531 (Japan)

Fax: (+81) 6-6850-6260, E-mail: kaneda@cheng.es.osaka-u.ac.jp

*Correspondence to Prof. Dr. K. Kaneda: E-mail: kaneda@cheng.es.osaka-u.ac.jp

Experimental

1) General

2) Catalyst preparation

   Preparation of metal oxide-supported noble metal catalysts

   Preparation of Ru/HAP and Ru/HT

   Preparation of Ru/SiO_2

3) Representative reaction procedures

   Typical reaction procedure under H_2 pressure

   Catalytic reactions in hexane solvent (Tables 2 and S4, entries 3, 4, 10, 22-24)

   Catalyst reuse experiments (Table S6)

   Isolation of the products (Tables 2 and S4, entries 3, 4, 10, 20)

4) References

Captions of Tables and Figures

- Table S1. Reported catalyst systems for the transformation of levulinic acid into 2-BuOH
- Table S2. Reported catalyst systems for the transformation of GVL into 2-BuOH
- Table S3. Reported catalyst systems for the selective transformation of stearic acid into *n*-heptadecane
- Table S4. Carbon-carbon scission of various oxygenates catalyzed by Ru/CeO_2
- Table S5. Curve fitting results of Ru K-edge EXAFS
- Table S6. Reuse experiments of Ru/CeO_2 catalyst
- Table S7. Support effects on Ru dispersion and 2-BuOH yields in the reaction of LA and 1,4-PeD

Captions of Figures

- Figure S1. Time profiles of the hydrogenative decarboxylation of LA using Ru/CeO_2. The reaction conditions were the same as those in Table 1, entry 1.
- Figure S2. The gas chromatography analysis of the gaseous phase after the reaction of LA in Table 1, entry 1.
- Figure S3. XRD patterns of (a) pristine CeO_2, (b) fresh Ru/CeO_2, (c) used Ru/CeO_2 without exposure to air, (d) used $Ce(OH)_3$ without exposure to air. Red triangle: $Ce(OH)_3$. Black triangle: CeO_2.
- Figure S4. Ru K-edge X-ray absorption experiments of the Ru catalysts. (a) Ru K-edge XANES spectra and (b) FT-spectra of Ru K-edge EXAFS of the Ru samples (Ru/CeO_2 fresh, Ru/CeO_2 used, Ru^0 powder, and RuO_2.
- Figure S5. Ce L_3-edge XANES spectra of Ru/CeO_2 (fresh and used), CeO_2 and $Ce_2(CO_3)_3$.
- Figure S6. FE-SEM images of (a) fresh Ru/CeO_2, (b) used Ru/CeO_2, and HR-TEM images of (c, d) used Ru/CeO_2. Yellow circles showed the presence of Ru nanoparticles.
- Figure S7. Plausible reaction pathway from LA to 2-BuOH catalyzed by RuNPs/$Ce(OH)_3$.

Experimental

1) General

Organic chemicals were purchased form Wako Pure Chemical Industries, Ltd, Tokyo Chemical Industry Co., Ltd and Sigma-Aldrich.

ZrO_2, hydroxyapatite, and $Pd(NO_3)_2$ nitrate were purchased from Wako Pure Chemical Industries, Ltd. $CeCl_3{\cdot}7H_2O$ was purchased from Sigma-Aldrich. $RuCl_3$, H_2PtCl_6, $IrCl_3$, and $RhCl_3$ were obtained from N.E. Chemcat. CeO_2 (JRC-CEO-2), TiO_2 (JRC-TIO-2), MgO (JRC-MGO-3), and γ-Al_2O_3 (JRC-ALO-8) were supplied by the Catalysis Society of Japan as reference catalysts. SiO_2 (CARiACT Q-3) was purchased from Fuji Silysia Chemical Ltd. Hydrotalcite was obtained from Tomita Pharmaceuticals. The following materials were prepared according to literature procedures: 1,3,5-pentanetriol[S1] and $Ce(OH)_3$.[S2] $Ce(OH)_3$ was handled under the Ar atmosphere.

Gas chromatography-mass spectrometry (GC-MS) analyses were performed on a Shimadzu QP-2010SE instrument equipped with a capillary column (InertCap WAX-HT, GL Science, 30 m × 0.25 mm i.d.) using diethyleneglycol dimethylether as the internal standard. Gaseous products were analyzed on a Shimadzu GC-2010 Plus instrument with a dielectric barrier discharge ionization detector (BID) equipped with a packed column (MICROPACKED ST, Shinwa Chemical Industries Ltd, 2.0 m × 1.0 mm i.d.). In situ IR spectra were recorded on a JASCO FT/IR-4100 instrument equipped with an MCT detector.

Ru *K*-edge and Ce L_3-edge X-ray absorption spectra were recorded at 298K in transmittance and fluorescence modes using Si (311) and Si (111) monochromators for Ru *K*-edge and Ce L_3-edge, respectively, on the 14B2 and 01B1 beam line stations at the SPring-8, Japan Atomic Energy Research Institute (JASRI), Harima, Japan (2015B1571, 2016A1103, 2016A1525, 2016B1546, 2016B1851, and 2017A1582). Data analysis was performed using Demeter ver. 0.9.21.[S3] Powder X-ray diffraction (XRD) patterns were acquired using a Philips X′Pert-MPD instrument with Cu-Kα radiation. Transmission electron microscopy (TEM) images were obtained using a Hitachi HF-2000 type microscope operating at 200 kV at the Research Center for Ultra-High Voltage Electron Microscopy, Osaka University. Inductively coupled plasma-atomic emission spectroscopy (ICP-AES) data were obtained using a SII Nano Technology SPS7800 instrument.

2) Catalyst preparation

Preparation of metal oxide-supported noble metal catalysts

The Ru/CeO_2 catalyst was prepared by the deposition‐precipitation method. CeO_2 (1 g) was added to 50 mL of an aqueous solution of $RuCl_3$ (4 mM). After stirring for 1 h, 3 mL of an

aqueous NH_3 solution (28%) was added to the reaction mixture, which was further stirred at 298 K for 12 h. The obtained slurry was filtered and washed with deionized water and dried at 383 K for 12 h, and finally calcined at 573 K for 3 h under static air atmosphere to obtain Ru/CeO_2 as a blown powder. ICP-AES analysis determined the Ru content in Ru/CeO_2 to be 2.0 wt%. Other metal oxide-supported Ru catalysts (Ru/ZrO_2, Ru/TiO_2, Ru/MgO, and Ru/Al_2O_3) were also prepared by the deposition‑precipitation method. M/CeO_2 (M = Pt, Pd, Rh, and Ir) catalysts were prepared in a similar way using H_2PtCl_6, $Pd(NO_3)_2$, $RhCl_3$, and $IrCl_3$ as the metal precursors.

Preparation of Ru/HAP and Ru/HT

Ru/HAP and Ru/HT were prepared according to reported procedures with slight modifications.[S4,S5] HAP or HT (1 g) was added to 50 mL of an aqueous solution of $RuCl_3$ (4 mM). After stirring for 12 h, the obtained slurry was filtered, washed with deionized water, and dried at 383 K for 12 h to obtain Ru/HAP or Ru/HT.

Preparation of Ru/SiO_2

Ru/SiO_2 was prepared by the impregnation method. SiO_2 (1 g) was added to 50 mL of an aqueous solution of $RuCl_3$ (4 mM) and the mixture was stirred magnetically for 4 h. Water was removed by rotary evaporation under reduced pressure to give a blown solid. The obtained powder was dried at 383 K for 12 h, then calcined at 573 K for 3 h under static air atmosphere to obtain Ru/SiO_2 as a black powder.

3) Representative reaction procedures

Typical reaction procedure under H_2 pressure

The reactions with levulinic acid were carried out in a 50 mL stainless steel autoclave equipped with a Teflon® vessel. The vessel was charged with 1 mmol of levulinic acid, 0.1 g of catalyst, and 3 mL of water; a Teflon®-coated magnetic stir bar was also added. The reactor was sealed, purged three times with H_2 at 1 MPa, then pressurized to 3 MPa, heated to 423 K, and stirred at 700 rpm for 12 h. After the reaction, the autoclave was cooled in an ice-water bath and the hydrogen gas was carefully released. The resulting reaction mixture was analyzed by GC-MS.

Catalytic reactions in hexane solvent (Tables 2 and S4, entries 3, 4, 10, 22-24)

Ru/CeO_2 (0.1 g) was pre-reduced in 3 mL of water under H_2 3 MPa, 423 K for 12 h using a 50 mL stainless steel autoclave. The obtained catalyst, Ru/CeO_2-reduced, was dried in vacuo. Then, the vessel was charged with 1 mmol of substrate, 0.1 g of Ru/CeO_2-reduced, and 3 mL of hexane; a Teflon®-coated magnetic stir bar was also added. The reactor was sealed, purged three times with H_2 at 1 MPa, then pressurized to 3 MPa, heated to 453 K, and stirred at 700 rpm for 12 h. After the reaction, the autoclave was cooled in an ice-water bath and the hydrogen gas was carefully released. The resulting reaction mixture was analyzed by GC-MS.

Catalyst reuse experiments (Table S6)

Reuse experiments were carried out after each hydrodecarboxylation of LA, in which the catalyst was separated from the reaction mixture by centrifugation, washed with water, and dried at 383 K overnight. The recovered catalyst was subsequently calcined in air for 3 h at 573 K, after which it was reused for the next reaction.

Isolation of the products (Tables 2 and S4, entries 3, 4, 10, and 20)

After the reaction, the autoclave was cooled in an ice-water bath and the hydrogen gas was carefully released. The solid catalyst was removed by filtration and washed with ether. The obtained liquid phase was extracted with ether. The organic phase was dried with anhydrous Na_2SO_4 and then, concentrated by rotary evaporation. In the case of the reactions using hexane solvent, the catalyst was removed by filtration and evaporation of the solvent afforded the desired products.

4) References

[S1] Utech, T., Köhler, J., & Bünsch, B. *Eur. J. Med. Chem*. **46**, 2157–2169 (**2011**).

[S2] Wang, L., Luan, Q., Yang, D., Yao, X., & Zhou, K. *RSC Adv*. **3**, 6339–6342 (**2013**).

[S3] Ravel, B., Newville, M. *J. Synchrotron Rad*. **12**, 537–541 (**2005**).

[S4] Yamaguchi, K., Mori, K., Mizugaki, T., Ebitani, K., Kaneda, K. *J. Am. Chem. Soc*. **122**, 7144–7145 (**2000**).

[S5] Motokura, K., Nishimura, D., Mori, K., Mizugaki, T., Ebitani, K., & Kaneda, K. *J. Am. Chem. Soc*. **126**, 5662–5663 (**2004**).

Table S1. Reported catalyst systems for the transformation of levulinic acid into 2-BuOH

Catalyst	Temp. [K]	H_2 pressure	Solvent	Conv. [%]	Yield [%]	TOF* [h^{-1}]	Ref.
**Ru/CeO_2**	**423**	**H_2 (3 MPa)**	**Water**	**>99**	**85**	**3.5**	**This work**
Ru/TiO_2	433	H_2 (15 MPa)	Water	100	61	5.1	S6
Ru/C	523	H_2 (40 MPa)	2-PrOH	100	25	18.1	S7
Ru-Re/C	433	H_2 (15 MPa)	Water	>99	3	2.6	S8

* TOF was calculated as the formation rate of 2-BuOH (2-BuOH mol/Ru mol h^{-1}).

[S6] Cabiac, A., Guillon, E., Pinel, C., Demailly, L. C., & Besson, M. French Patent, FR 3008970A1, 30 Jan (**2015**).

[S7] Obregón, I., Gandarias, I., Al-Shaal, M. G., Mevissen, C., Arias, P. L., Palkovits, R., & Gandarias, I. *ChemSusChem* **9**, 2488–2495 (**2016**).

[S8] Corbel-Demailly, L., Ly, B. K., Minh, D. P., Tapin, B., Especel, C., Epron, F., Cabiac, A., Guillon, E., Besson, M. & Pinel, C. *ChemSusChem* **6**, 2388–2395 (**2013**).

Table S2. Reported catalyst systems for the transformation of GVL into 2-BuOH

Catalyst	Temp. [K]	H_2 pressure	Solvent	Conv. [%]	Yield [%]	TOF* [h^{-1}]	Ref.
**Ru/CeO_2**	**423**	**H_2 (3 MPa)**	**Water**	**>99**	**83**	**3.5**	**This work**
Ru/C	463	H_2 (40 MPa)	2-PrOH	97	37	8.6	S7
Ru/C	463	H_2 (100 MPa)	Neat	>99	36	6.4	S9

[S9] Al-Shaal, M. G., Dzierbinski, A., Palkovits, R. *Green Chem.* **16**, 1358–1364 (**2014**).

Table S3. Reported catalyst systems for the selective transformation of stearic acid into *n*-heptadecane

Catalyst	Temp. [K]	H_2 pressure	Solvent	Conv. [%]	Yield [%]	Ref.
**Ru/CeO_2**	**453**	**H_2 (3 MPa)**	***n*-Hexane**	**>99**	**97**	**This work**
$Ru/La(OH)_3$	473	H_2 (4 MPa)	*n*-Dodecane	100	98	S10
Ni/ZrO_2	533	H_2 (4 MPa)	*n*-Dodecane	100	96	S11
Ni/SiO_2	573	H_2 (3 MPa)	*n*-Dodecane	100	96	S12
$Ni/\gamma\text{-}Al_2O_3$	573	H_2 (3 MPa)	*n*-Dodecane	100	96	S12
Ni/CeO_2	533	H_2 (4 MPa)	*n*-Dodecane	100	93	S11
Pd-Ni/HZSM5	553	H_2 (0.2 MPa)	Limonene	100	92	S13
Pd/Al_2O_3	623	H_2 (1.4 MPa)	*n*-Dodecane	>99	91	S14
$Ni/\gamma\text{-}Al_2O_3$	543	H_2 (0.8 MPa)	*n*-Dodecane	>99	90	S15
Ru/NCNT-800	498	H_2 (4 MPa)	*n*-Hexane	>99	87	S16
Ru/HAP	473	H_2 (2 MPa)	Water	>99	78	S17

[S10] Guo, J., Xu, G., Shen, F., Fu, Y., Zhang, Y., & Guo, Q. *Green Chem*. **17**, 2888–2895 (**2015**).

[S11] Peng, B., Yuan, X., Zhao, C., & Lercher, J. A. *J. Am. Chem. Soc.* **134**, 9400–9405 (**2012**).

[S12] Hachemi, I., Jeništová, K., Mäki-Arvela, P., Kumar, N., Eränen, K., Hemming, J., & Murzin, D. Y. *Catal. Sci. Technol*. **6**, 1476–1487 (**2016**).

[S13] Zhang, J., Zhao, C. *ACS Catal*. **6**, 4512−4525 (**2016**).

[S14] Berenblyum, A. S., Podoplelova, T. A., Shamsiev, R. S., Katsman, E. A., & Danyushevsky, V. Ya. *Pet. Chem*. **51**, 336–341 (**2011**).

[S15] Kumar, P., Yenumala, S. R., Maity, S. K., Shee, D. *Appl. Catal. A* **471**, 28–38 (**2014**).

[S16] Li, J., Wang, S., Liu, H.-Y., Zhou, H.-j., & Fu, Y. *ChemistrySelect* **2**, 33–41 (**2017**).

[S17] Xu, G., Zhang, Y., Fu, Y., & Guo, Q. *ACS Catal*. **7**, 1158–1169 (**2017**).

Table S4. Carbon-carbon scission of various oxygenates catalyzed by Ru/CeO_2[a]

Entry	Substrate	Temp [K]	Time [h]	Conv. [%] [b]	Yields [%] [b]			
	**carboxylic acid**							
1	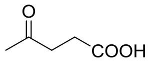	423	12	>99	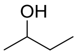	85	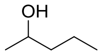	5
2	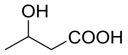	433	9	>99	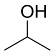	81	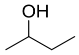	4
3 [c]	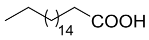	453	48	>99	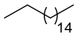	(97)		
4 [c]	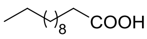	453	48	>99	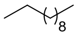	(87)		
5	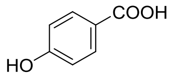	433	12	>99	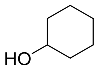	75	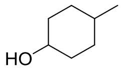	3
6	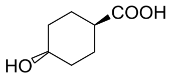	433	24	>99	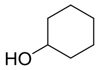	76	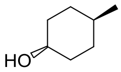	6
	**esters**							
7	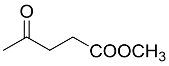	423	12	>99	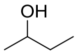	82	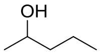	5
8	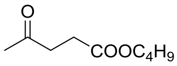	433	12	>99	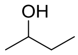	82	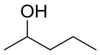	5
9	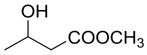	433	9	>99	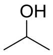	82	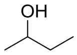	4
10 [c]	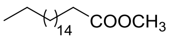	453	48	>99	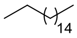	(96)		
11	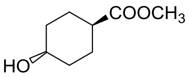	433	24	>99	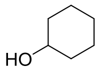	68	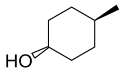	5
	**lactones**							
12	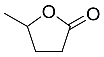	423	12	>99	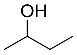	83	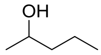	5
13	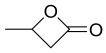	433	12	>99	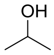	86	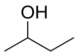	6
14	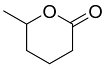	433	18	>99	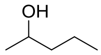	80	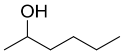	4

Table S4. Continued.

Entry	Substrate	Temp [K]	Ttime [h]	Conv. [%] [b]	Yields [%] [b]			
	**polyols**							
15	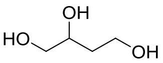	433	12	>99	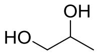	72	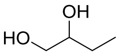	2
16	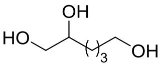	433	12	>99	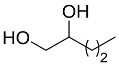	72	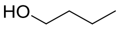	11
17	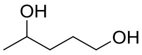	423	12	>99	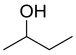	97	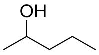	3
18	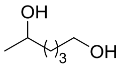	433	12	>99	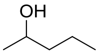	92	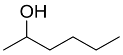	trace
19	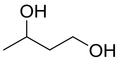	423	12	>99	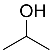	92	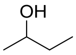	4
20 [d]	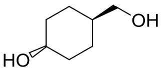	433	60	>99	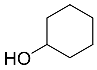	(92)		
21	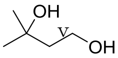	433	9	>99	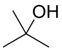	93	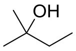	3
	**mono-ols**							
22 [e]	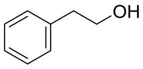	453	12	>99	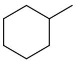	89	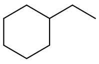	4
23 [e]	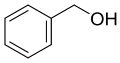	453	12	>99	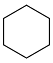	95	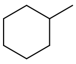	3
24 [e]	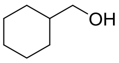	453	24	>99	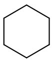	97	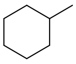	2
25	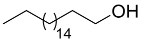	453	24	>99	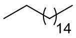	93	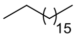	trace
26	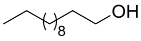	453	9	>99	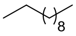	99	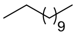	trace
27	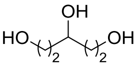	433	12	>99	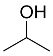	82	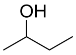	14

[a] Reaction conditions: substrate (1 mmol), Ru/CeO_2 (0.1 g, Ru: 2 mol%), water (3 mL), H_2 (3 MPa).

[b] Determined by GC-MS using the internal standard technique. The values in parentheses are isolated yields.

[c] Reaction conditions: substrate (4 mmol), Ru/CeO_2 (0.3 g, Ru: 1.5 mol%), *n*-hexane (15 mL), H_2 (3 MPa).

[d] Reaction conditions: substrate (4 mmol), Ru/CeO_2 (0.5 g, Ru: 2.5 mol%), water (15 mL), H_2 (3 MPa).

[e] Reaction conditions: substrate (1 mmol), Ru/CeO_2 (0.1 g, Ru: 2 mol%), *n*-hexane (3 mL), H_2 (3 MPa).

Table S5. Curve fitting results of Ru K-edge EXAFS

Catalyst	Shell	CN[a]	R [Å] [b]	DW [Å] [c]	Dispersion[d]	d_{av} [nm] [e]
Ru/CeO_2 fresh	Ru-O	5.6	2.00	0.083	-	-
Ru/CeO_2 used	Ru-Ru	6.6	2.67	0.073	48%	2.8
Ru powder	Ru-Ru	12.0	2.67	0.070	-	-
RuO_2	Ru-O	6.0	1.98	0.010	-	-

[a] CN: coordination number. [b] R: bond distance. [c] DW: Debye-Waller factor.

[d] Estimated by CO chemisorption. [e] Average particle size estimated from dispersion.

Table S6. Reuse experiments of Ru/CeO_2 catalyst

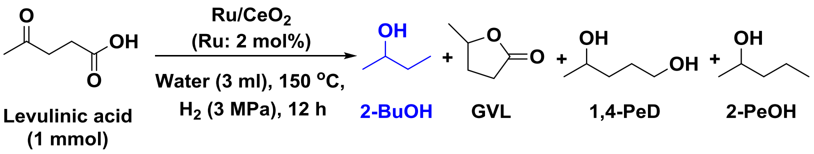

Entry	Catalyst	Conv.	Yield [%][a]			
		[%][a]	2-BuOH	GVL	1,4-PeD	2-PeOH
1	Run 1	>99	85	0	0	5
2	Run 2	>99	83	0	0	5
3	Run 3	>99	82	0	0	5
4	Run 4	>99	73	6	7	2
5	Run 5	>99	73	4	4	3

a Conversion and yields were determined by GC-MS using an internal standard technique.

The reaction conditions were the same as that of in Table 1, entry 1.

Table S7. Support effects on Ru dispersion and 2-BuOH yields in the reaction of LA and 1,4-PeD

Entry	Catalyst	Ru dispersion [%] [a]	2-BuOH Yield from LA [%] [b]	2-BuOH Yield from 1,4-PeD [%] [c]
1	Ru/CeO_2	48	85	97
2	Ru/ZrO_2	30	57	74
3	Ru/HT	16	17	38
4	Ru/SiO_2	0.8	6	24
5	Ru/MgO	3.2	6	20
6	RuAl_2O_3	3.0	1	tr.

[a] Calculated from CO chemisorption.

[b] The data were also showed in Table 1.

[c] The reaction conditions were similar to those in Table 2, entry 17.

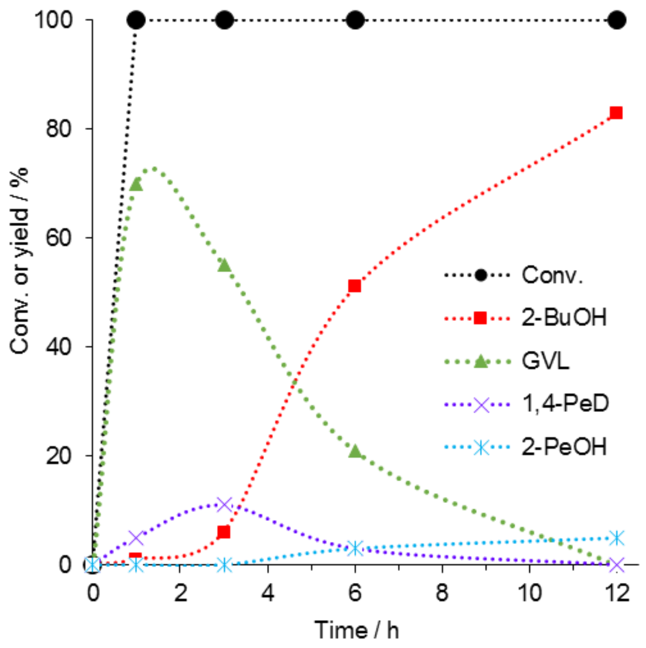

Figure S1. Time profiles of the hydrogenative decarboxylation of LA using Ru/CeO_2. The reaction conditions were the same as those in Table 1, entry 1.

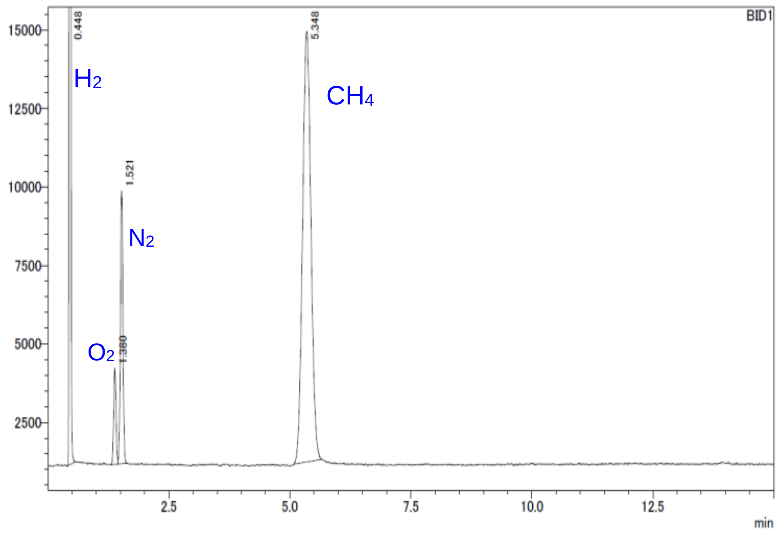

Figure S2. The gas chromatography analysis of the gaseous phase after the reaction of LA in Table 1, entry 1.

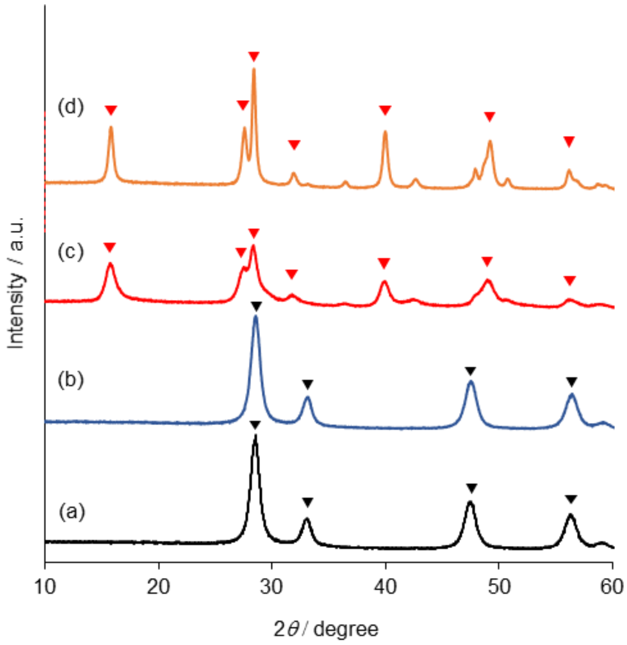

Figure S3. XRD patterns of (a) pristine CeO_2 (JCPDS card no. 34–0394), (b) fresh Ru/CeO_2, (c) used Ru/CeO_2 without exposure to air, and (d) $Ce(OH)_3$ without exposure to air (JCPDS card no. 74-0665). Red triangle: $Ce(OH)_3$. Black triangle: CeO_2.

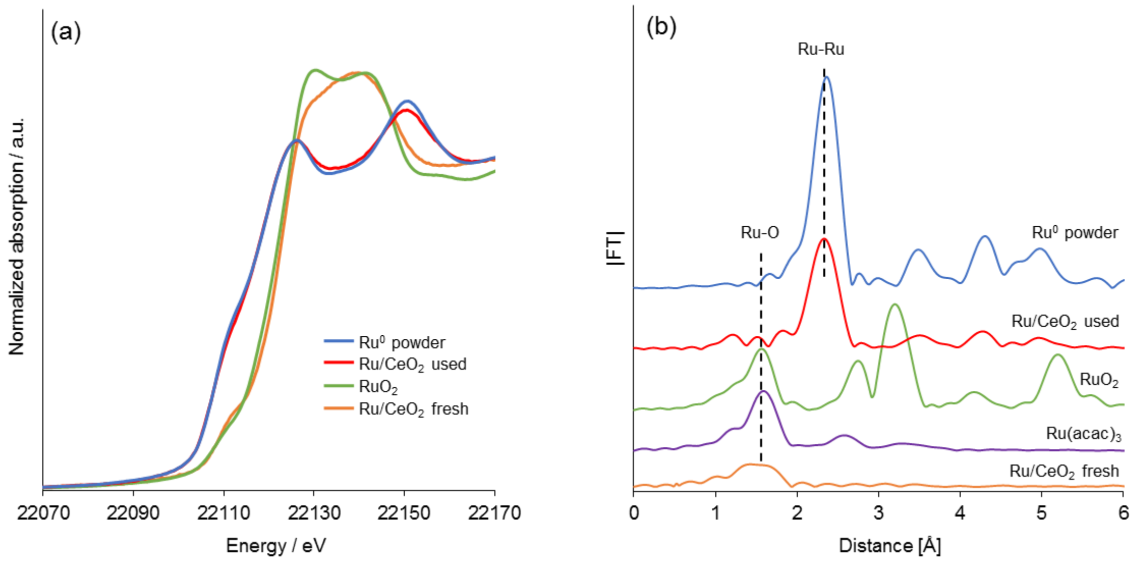

Figure S4. Ru K-edge X-ray absorption experiments of the Ru catalysts. (a) Ru *K*-edge XANES spectra and (b) FT-spectra of Ru *K*-edge EXAFS of the Ru samples (Ru/CeO2 fresh, Ru/CeO_2 used, Ru^0 powder, and RuO_2.

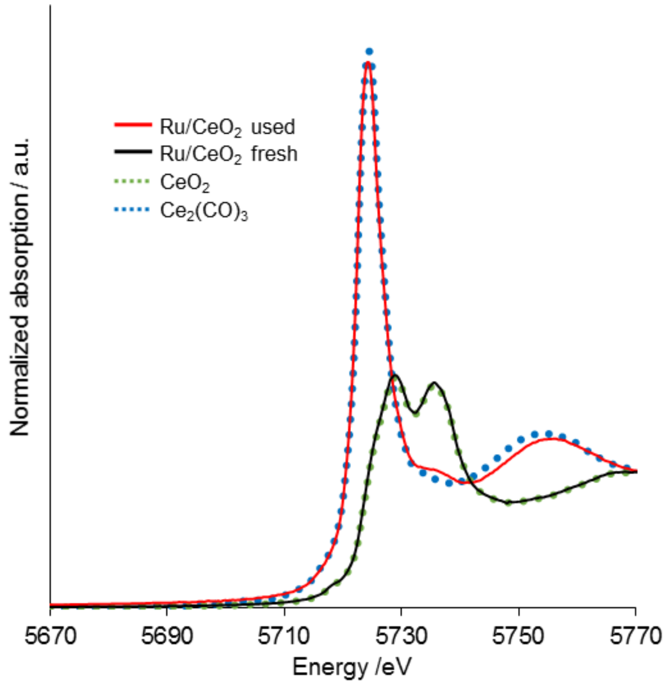

Figure S5. Ce L_3-edge XANES spectra of Ru/CeO_2 (fresh and used), CeO_2 and $Ce_2(CO_3)_3$.

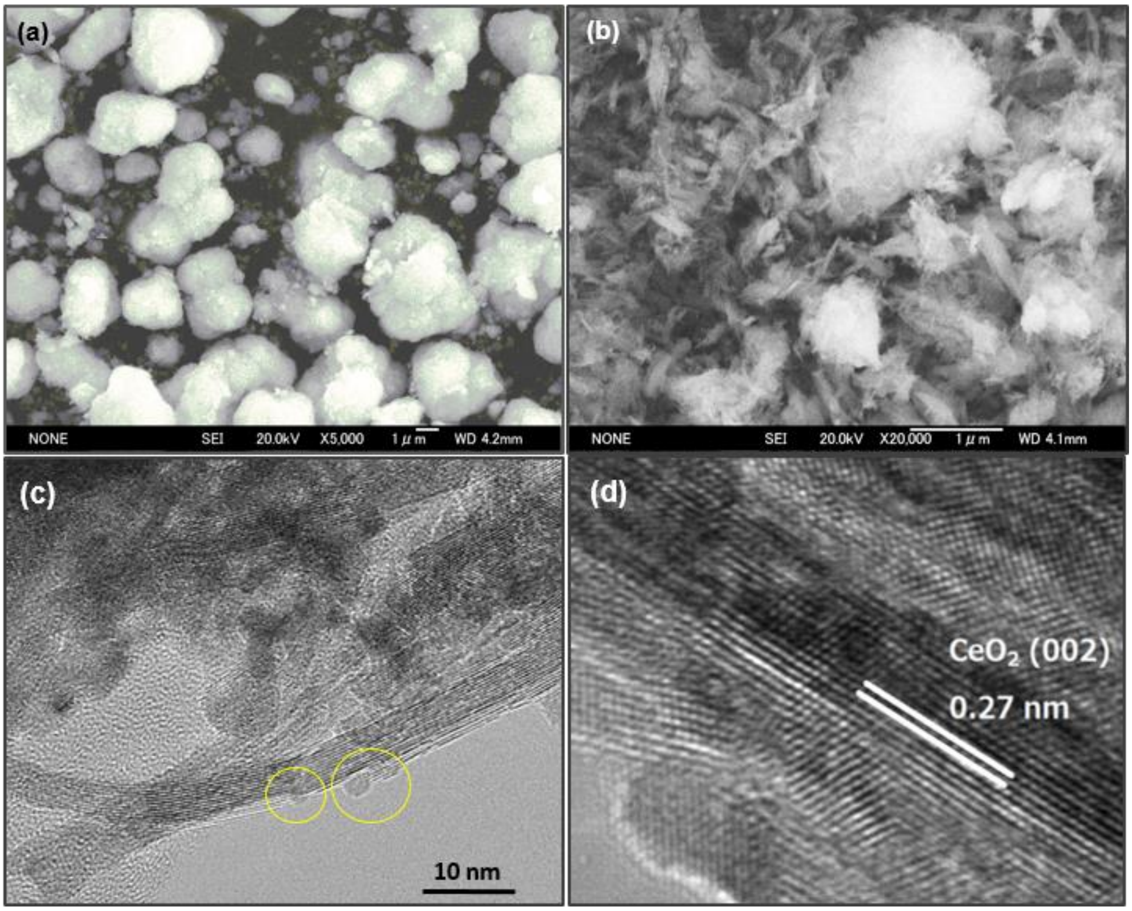

Figure S6. FE-SEM images of (a) fresh Ru/CeO_2, (b) used Ru/CeO_2, and HR-TEM images of (c, d) used Ru/CeO_2. Yellow circles showed the presence of Ru nanoparticles..

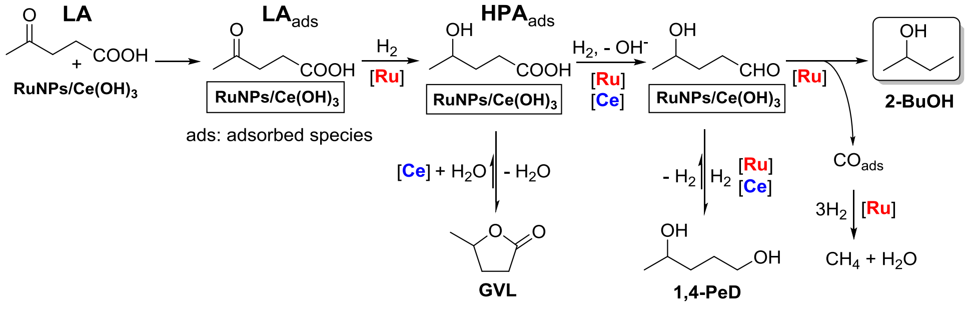

Figure S7. Plausible reaction pathway from LA to 2-BuOH catalyzed by RuNPs/$Ce(OH)_3$.
